# Color compatibility between dental structures and three different types of ceramic systems

**DOI:** 10.1186/s12903-021-01404-7

**Published:** 2021-02-17

**Authors:** Ioana-Sofia Pop-Ciutrila, Razvan Ghinea, Horatiu A. Colosi, Javier Ruiz-López, Maria M. Perez, Rade D. Paravina, Diana Dudea

**Affiliations:** 1 Department of Conservative Dentistry and Endodontics, Faculty of Dental Medicine, Iuliu Hatieganu University of Medicine and Pharmacy, 33 Motilor Street, 400001 Cluj-Napoca, Romania; 2 Department of Optics, Faculty of Science, University of Granada, Campus de Fuentenueva, s/n, 18071 Granada, Spain; 3 Houston Center for Biomaterials and Biomimetics (HCBB), University of Texas School of Dentistry at Houston, 7500 Cambridge St., Ste. 5350, Houston, TX USA; 4 Department of Physics, Faculty of Sciences, University of Craiova, 13 AI Cuza Street, 200585 Craiova, Romania; 5 Department of Medical Informatics and Biostatistics, Faculty of General Medicine, Iuliu Hatieganu University of Medicine and Pharmacy, 6 Louis Pasteur Street, 400349 Cluj-Napoca, Romania; 6 Department of Restorative Dentistry and Prosthodontics, Houston Center for Biomaterials and Biomimetics (HCBB), University of Texas School of Dentistry at Houston, 7500 Cambridge St., Ste. 5350, Houston, TX USA; 7 Department of Prosthodontics and Dental Materials, Iuliu Hatieganu University of Medicine and Pharmacy, 32 Clinicilor Street, 400006 Cluj-Napoca, Romania

**Keywords:** Color coordinates, Coverage error, Dental ceramics, Dentine, Enamel, Spectroradiometer

## Abstract

**Background:**

To assess color compatibility between dental structures (human enamel and dentine) and three different types of ceramic systems.

**Methods:**

Samples (1 and 2 mm-thick) of extracted tooth (containing dentine and enamel areas) and three ceramic systems with different shades and opacities (HT–High Translucent, T–Translucent) were prepared for this study: Vita Suprinity—VS (HT, T; A1, A2, A3, A3.5, B2, C2, D2) (Vita Zahnfabrik); Vita Enamic—VE (HT, T; 1M1, 1M2, 2M2, 3M2) (Vita Zahnfabrik) and Noritake Super Porcelain EX-3—NKT (A1, A2, A3, A3.5, B2, C2, D2) (Kuraray Noritake Dental). Reflectance measurements of all samples were performed over black backgrounds using a non-contact spectroradiometer (SpectraScan PR-670, Photo Research) under a CIE 45°/0° geometry. CIE L*a*b* color parameters were measured and CIELAB/CIEDE2000 color differences (ΔE_00_/ΔE^*^_ab_) and corresponding Coverage Error (CE) of ceramic system for dentine or enamel samples were calculated. Color data was analyzed using one-way ANOVA and post-hoc multiple comparisons tests. CE values were interpreted by comparisons with available 50:50% acceptability color threshold (AT) for dentistry.

**Results:**

Statistically significant differences in lightness were found among all ceramic systems and human dentine (p < 0.001), while no significant differences were registered between enamel and VSHT, T and VEHT. 1 mm dentine showed no statistical differences with VST and VSHT for a* coordinate, while 2 mm dentine showed no significant differences (p > 0.05) with VEHT. Thin samples (1 mm) of dentine and enamel showed significant statistical differences (p < 0.05) for b* coordinate with less translucent materials (NKT, VET and VST). For dentine samples, none of the ceramic materials provided a CE lower than AT. VSHT provided the best CE for 1 mm-thick (CE_00_ = 1.7, CE_ab_ = 1.9) and for 2 mm-thick (CE_00_ = 2.3; CE_ab_ = 2.5) enamel samples.

**Conclusions:**

Color coordinates of evaluated esthetic ceramic systems were statistically different from those of human dentine in almost all cases. The evaluated ZrO_2_ lithium silicate glass–ceramic (VS), with its two levels of translucency, provided lower CE values with human enamel samples while conventional feldspathic ceramic (NKT) and hybrid ceramic systems (VE) demonstrated a better color compatibility with dentin samples.

## Background

The wide range of available all-ceramic systems, with ever improving mechanical and optical properties, are trying to reproduce, to a great extent, the properties of natural dental structures [1, 2].

The complexity of tooth color, however, seems difficult to achieve by any existing restorative material. Light reaching tooth surface is partly absorbed, diffused, transmitted or reflected by hard dental structures. Recent research revealed that these physical optical phenomena, occurring at the surface and inside tooth structures, are strongly influenced by the tooth type [3]. Nowadays, ceramic systems provide different opacities and a large palette of colors, aiming to cope with any clinical situation and to act as “biomimetic”, with similar optical and mechanical properties as those of the tissues being replaced. However, these materials must be carefully handled, as important color variations were observed between the same shades of different lot numbers and between brands with similar shade designation, which can influence the esthetic outcome of the final restoration [4].

Conventional feldspathic ceramic is considered to be the most translucent ceramic material, the “gold standard” in esthetic dentistry, but on the other hand is brittle and has a very low resistance to fracture [5, 6]. Successful esthetic outcomes in the reconstruction of anterior teeth are also atributed to glass–ceramics with leucite and lithium disilicate reinforced crystals [7]. It was proven that their mechanical and physical properties are better than those of classical feldspar ceramics [8].

Computer-aided design and computer-aided manufacturing techniques (CAD/CAM) have gained ground over the years. Using these procedures, several materials with large clinical indications, including zirconia, alumina, lithium silicate and disilicate based ceramics can be fast and easy milled [9–11]. Hybrid ceramics, made up of leucite based ceramic network reinforced by zirconia and interconected with an acrylate polymer network are also available as CAD/CAM blocks. It was proven that they have a higher damage tolerance and a lower elastic modulus, hardness and stiffness than other indirect restorative ceramic materials [12, 13]. However, when compared to glass–ceramics, feldspathic ceramics and resin composites, their translucency is lower [14].

Zirconia-reinforced lithium silicate glass–ceramic, containing more than 10% Zr has been recently introduced for aesthetic reconstruction of posterior teeth. This CAD/CAM processed material proved to be strong, fracture resistant and natural looking, having similar optical properties [10].

However, there is a limited number of studies that report on the color compatibility of these ceramics with the hard tissues they are supposed to replace. Therefore, it is essential to further investigate the optical and colorimetric properties of these materials, in comparison with the enamel and dentine, in an effort to understand their behavior and to provide a scientific support for clinical decision-making.

In esthetic dentistry, the choice of the most appriopiate material of restoration represent the keys to the succes of the final clinical result [15]*.* The optical color parameters as well as the translucency depend on the type of material and its thickness [16, 17]. In both research and clinical dentistry, colorimetric assessment of dental structures and materials is mainly done using the CIELAB color space and its associated total color difference formulas CIELAB ($\Delta {E}_{ab}^{*}$) and CIEDE2000 ($\Delta {E}_{00}$) [18, 19]. In the CIELAB tridimensional color representation system, each color is defined related to three colorimetric axes: L* (lightness—achromatic axis), a* (green–red axis), and b* (blue–yellow axis). In terms of color differences, even though significant correlations between the two formulas were observed [20], the CIEDE2000 color difference formula was considered to provide a better fit with human perception [21, 22].

The use of color differences in dental research is wide, covering diverse areas such as shade correspondence [23], instrumental shade matching [24], color difference thresholds [25] or performance assessment of dental shade guides through computation of Coverage Error [26]. The Coverage Error (CE) is a concept introduced in dentistry [26–29] to assess the color representation of specific shade guides (or restorative systems comprising several shades) to samples of human dental structures.

The main objective of this study was to test the color compatibility between dental structures (enamel and dentine) and three different types of ceramic systems, considered as representative for their class (ZrO_2_ lithium silicate machinable ceramic, hybrid ceramic and feldspathic ceramic). The null hypotheses tested were: (1) Color parameters of analysed ceramics did not differ from the corresponding values of human enamel and dentine; (2) the CE for enamel and dentine of all studied materials is below or at the clinically acceptable threshold for color in dentistry.

## Methods

### All-ceramic materials samples

Three different all-ceramic materials were evaluated (Table 1). A total of 132 square samples (3 per group) of approximately 1.2 and 2.2 mm thickness were fabricated from HT (high translucent) and T (translucent) Vita Suprinity (VSHT and VST, respectively) ingots and Vita Enamic (VEHT and VET, respectively) blocks (14 mm × 12 mm × 18 mm). The available shades were A1, A2, A3, A3.5, B2, C2 and D2 [according to the Vita Classical shade guide (Vita Zahnfabrik, Bad Säckingen, Germany)] for VSHT and VST and 1M1, 1M2, 2M2, 3M2 [according to the Vita 3D-Master shade guide (Vita Zahnfabrik, Bad Säckingen, Germany)] for VEHT and VET. For the Noritake Super Porcelain EX-3 (NKT) feldspathic ceramic, 42 round samples (3 per group) with 10 mm diameter and approximately 1.2 and 2.2 mm thickness were fabricated with shades A1, A2, A3, A3.5, B2, C2 and D2 (according to the Vita Classical shade guide system).

**Table 1:** Shades and opacities of all ceramic materials tested

Material	Manufacturer	Classification	Shade	Batch #	Thickness mm	Number of samples
Vita Suprinity	Vita Zahnfabrik, Bad Säckingen, Germany	Machinable ZrO_2_ lithium silicate	HT-A1	50740	1 (± 0.2) mm/2 (± 0.2) mm	42 n* = 3
			HT-A2	40331		
			HT-A3	50741		
			HT-A3,5	40180		
			HT-B2	51360		
			HT-C2	50840		
			HT-D2	41130		
			T-A1	51361	1 (± 0.2) mm/2 (± 0.2) mm	42 n* = 3
			T-A2	49141		
			T-A3	47558		
			T-A3.5	50920		
			T-B2	49341		
			T-C2	51361		
			T-D2	51650		
Vita Enamic	Vita Zahnfabrik, Bad Säckingen, Germany	Machinable ceramic reinforced by a polymer network	HT-1M1	47960	1 (± 0.2) mm/2 (± 0.2) mm	24 n* = 3
			HT-1M2	53720		
			HT-2M2	45810		
			HT-3M2	43180		
			T-1M1	44680	1 (± 0.2) mm/2 (± 0.2) mm	24 n* = 3
			T-1M2	34720		
			T-2M2	40501		
			T-3M2	48001		
Noritake Super Porcelain EX-3	Kuraray Noritake Dental, Tokyo, Japan	Conventional feldspathic	A1		1 (± 0.2) mm/2 (± 0.2) mm	42 n* = 3
			A2			
			A3			
			A3,5			
			B2			
			C2			
			D2			

* refers to the number of samples per group of shade and thickness

**Fig. 1:**
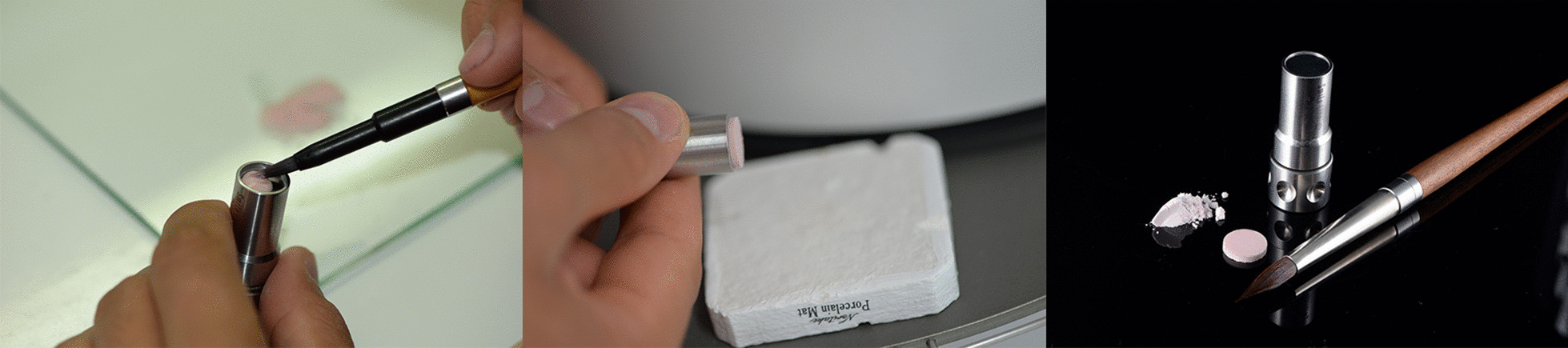
Manufacturing of feldspathic ceramic samples

The machinable ceramic samples were obtained by cutting CAD/CAM blocks with a water-cooled diamond disk at low speed in a precision saw machine (Isomet 1000; Buehler, Lake Bluff, IL, USA). Zirconia reinforced lithium silicate glass–ceramic samples were sliced in their precrystallized condition. The slices were then submitted to a crystallization process in a furnace (Programat EP 3000; Ivoclar Vivadent, Schaan, Liechtenstein), according to the manufacturers’ instructions. The conventional feldspathic ceramic samples were fabricated with a porcelain sampler (Smile Line, St-Imier, Switzerland). This system facilitated the manufacturing of very precise round samples of a determined thickness (Fig. 1). The samples were fired according to the manufacturer’s guidelines.

All samples were finished and polished, under constant pressure by the same operator, on a grinder/polisher (Ecomet 30 Manual; Buehler, Lake Bluff, IL, USA) increasingly with wet 120-, 240-, 400-, 600-, 800-, and 1200-grit silicon carbide paper. The final thicknesses of all samples were established to 1 (± 0.2) mm and 2 (± 0.2) mm as determined with a digital micrometer (Powerfix Profi + ; OWIM, Haiger, Germany) with an accuracy of ± 0.05 mm.

### Human Enamel and Dentine samples

**Fig. 2:**
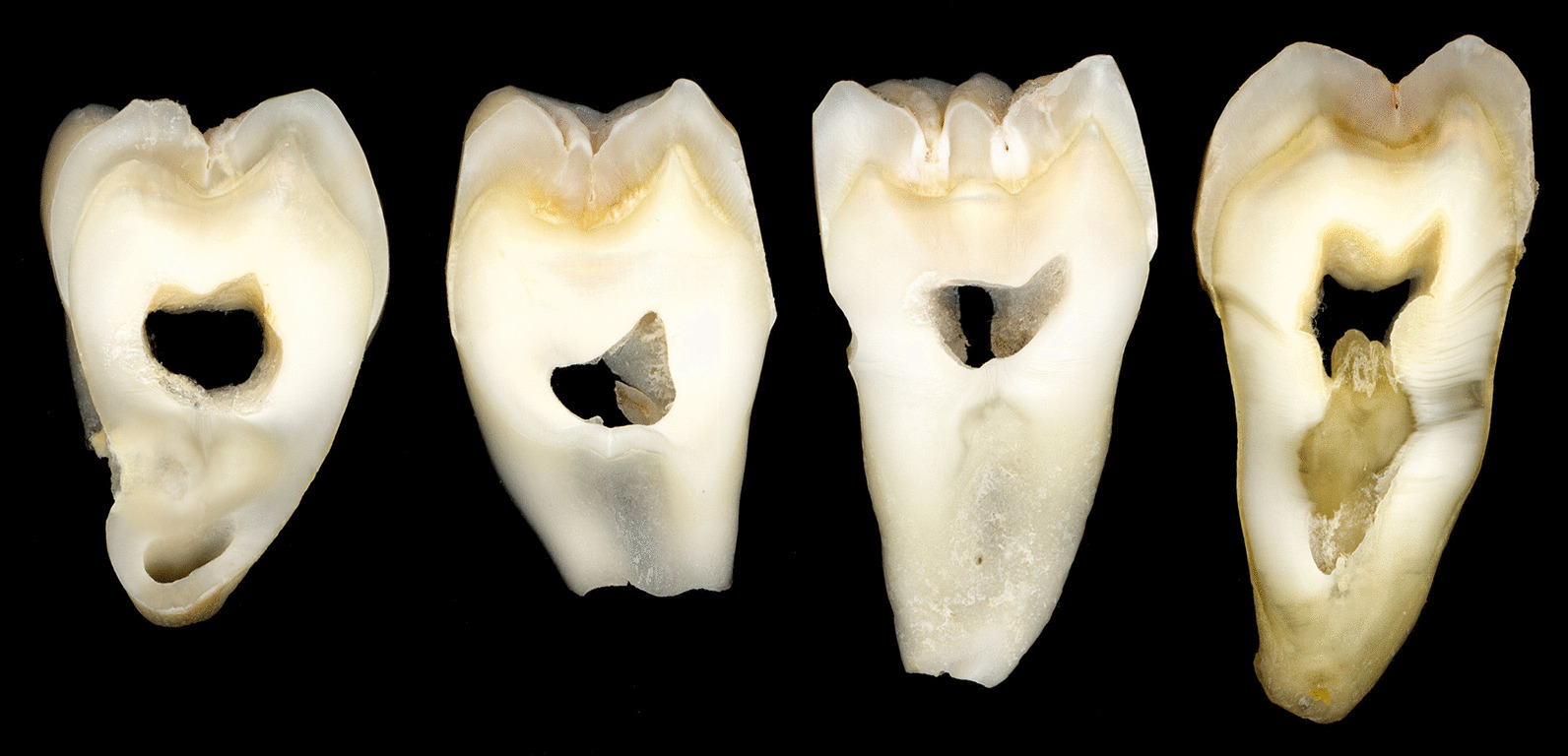
Tooth slices containing enamel and dentine substrate

Freshly extracted human posterior teeth obtained from a tissue bank were preserved in sodium azide solution (0.9% saline, 0.25% NaN3) prior to sectioning. Before cutting them, they were visually inspected and cleaned from debris and only those that were free of caries, restorations or any other pathological discolorations were included in the study. A low speed diamond saw (Isomet 1000; Buehler, Lake Bluff, IL, USA) was used to cut approximately 1.2 and 2.2 mm-thick sagittal bucco-lingual tooth slices, each containing both enamel and dentine substrate (Fig. 2). After the polishing and finishing procedures 1 mm (± 0.2) and 2 mm (± 0.2) thick hard tissue samples were obtained. The study protocol was approved by the Institutional Review Board (IRB) (HSC-DB-14-0744) prior to the beginning of the study.

The 174 ceramic samples and the 32 tooth slices of 2 different thicknesses were cleaned in an ultrasonic bath (Elmasonic S30H; Elma Schmidbauer, Singen, Germany) with distilled water for 10 min and dried under compressed air before they were measured.

### Spectroradiometric measurements

**Fig. 3:**
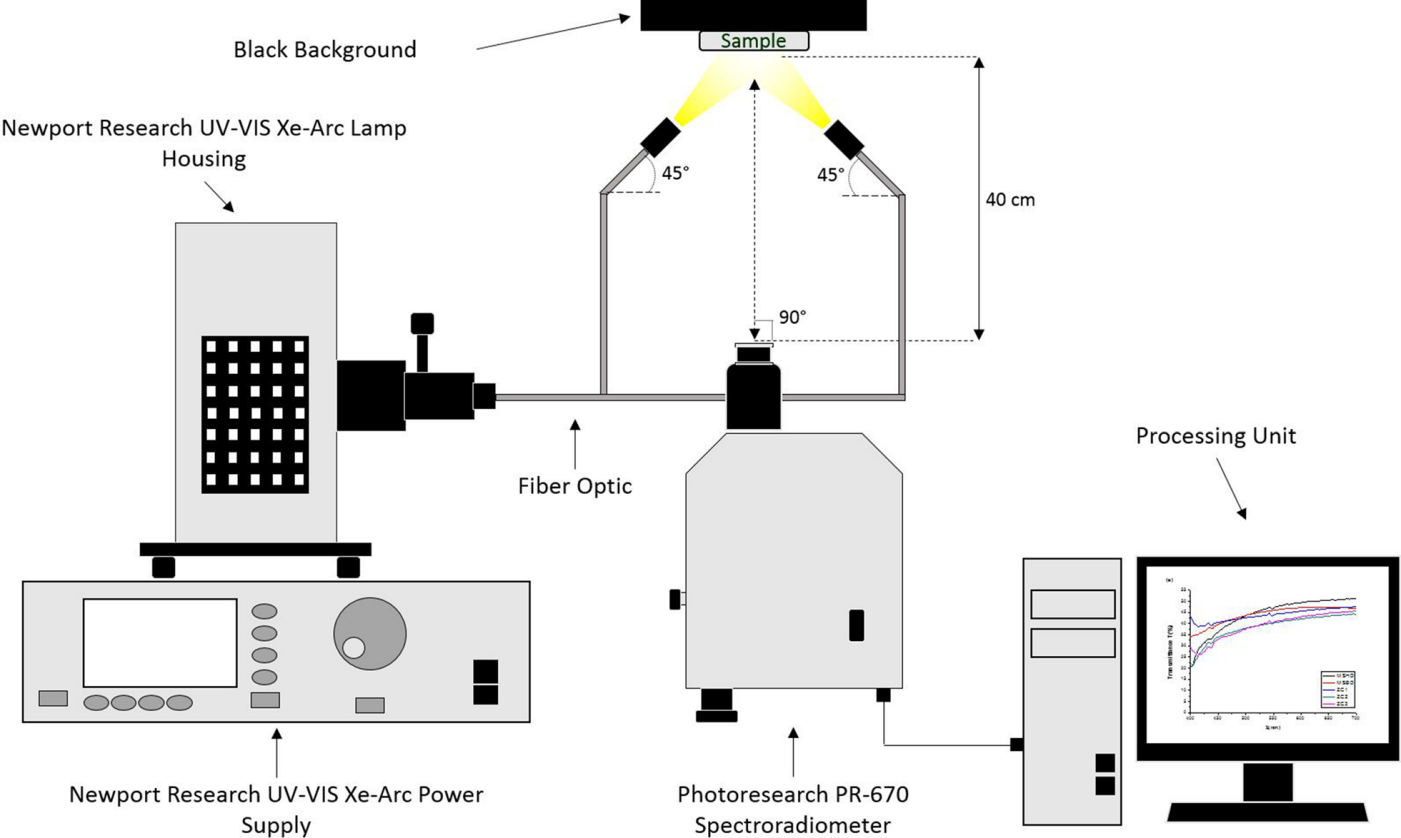
Experimental set-up used for objective color measurements of the samples

Three short-term repeated reflectance measurements in the 380–780 nm range (without replacement; over a black background) were performed for each sample inside a completely dark room using a spectroradiometer (SpectraScan PR 670; Photo Research, Syracuse, NY, USA), a fiber-coupled Xe-Arc light source (66485-300; Newport Corporation, Irvine, CA, USA) and a Spectrally Calibrated Reflectance Standard (SRS-3; Photo Research, Syracuse, NY, USA). Between the samples and the black background, a saturated sucrose solution (refractive index n = 1.5 approximately) was interposed. The spectroradiometer was placed 40 cm away from the samples and the illuminating/measuring geometry corresponded to CIE 45°/0° (Fig. 3). Spectral reflectance values were converted into CIE L*a*b* color coordinates using the CIE 2º Standard Observer and the CIE D65 Standard Illuminant.

### Color differences and coverage error computation

The color differences between shade samples of each ceramic systems and each dentine or enamel sample (n = 16) were calculated using both the CIELAB ($\Delta {E}_{ab}^{*}$) and CIEDE2000 ($\Delta {E}_{00}$) color difference formulas [18, 19]:

1 $$\Delta {E}_{ab}^{*} = \sqrt{{(\Delta {L}^{*})}^{2}+{(\Delta {a}^{*})}^{2}+{(\Delta {b}^{*})}^{2},}$$

where ΔL*, Δa* and Δb* are the differences in the respective coordinates for a pair of samples.

2 $$\Delta {E}_{00}={[({\frac{\Delta L\mathrm{^{\prime}}}{{K}_{L}{S}_{L}})}^{2}+({\frac{\Delta C\mathrm{^{\prime}}}{{K}_{C}{S}_{C}})}^{2}+({\frac{\Delta H\mathrm{^{\prime}}}{{K}_{H}{S}_{H}})}^{2}+{R}_{T}\left(\frac{\Delta C\mathrm{^{\prime}}}{{K}_{C}{S}_{C}}\right)\left(\frac{\Delta H\mathrm{^{\prime}}}{{K}_{H}{S}_{H}}\right)]}^{1/2}$$

where ΔL′, ΔC′ and ΔH′ are the differences in lightness, chroma and hue for a pair of samples in CIEDE2000.

Coverage error (CE) was calculated as the mean value of the minimal color differences (${\Delta E}_{min}$) among the color of dentine or enamel samples and the color representatives (shades) of each ceramic system, as follows [27]:

3 $$\mathrm{CE} = \sum \frac{{\Delta E}_{min}}{n},$$

where ${\Delta E}_{min}$ was expressed as $\Delta {E}_{ab}^{*}$ or $\Delta {E}_{00}$, depending on the color difference formula used, and $n$ represents the number of dentine or enamel samples.

CE values were compared with clinically relevant 50:50% Perceptibility Thresholds—PT—(ΔE_00_ = 0.8; ΔE^*^_ab_ = 1.2) and 50:50% Acceptability Thresholds—AT—(ΔE_00_ = 1.8; ΔE^*^_ab_ = 2.7), as reported in a multi-center study [25].

### Statistical analysis

To describe the variability of measured color parameters CIE L*,a*, and b*, the 95% confidence intervals for means and standard deviations (SD) of these variables have been computed for all investigated enamel, dentine and ceramic samples. Confidence intervals for mean CIE L*,a*, and b* have also been bootstrapped based on replications of 1000 samples. The normality of the measured color parameters has been investigated using Kolmogorov–Smirnov, Shapiro–Wilk tests and Quantile–Quantile Plots and the homogeneity of variance was assessed by Levene’s test. One-way ANOVA was used to compare the mean values of L*,a*, and b* color coordinates for all ceramic and tooth samples of different opacities, shades and thicknesses. Multiple comparisons using Tamhane’s error correction procedure have been performed for post-hoc comparisons of the measured color parameters between enamel, dentine and ceramic samples. The level of statistical significance has been set at α = 0.05 (SPSS Statistics 20, IBM Armonk, New York, USA).

## Results

Means, standard deviations and results of statistical analysis of CIE L*a*b* color coordinates of the evaluated materials for the two analysed thicknesses are presented in Table 2. The lightness mean values (CIE L*) of all materials ranged from 65.61 to 86.89 units for 1 mm thick samples and from 66.54 to 82.67 units for 2 mm-thick samples. Human dentine exhibited the highest lightness mean values among all materials studied, being more yellowish and reddish at the same time, independently of thickness. The decreasing order of mean CIE L* value for all studied materials was: dentine > VET > NKT > VST > VEHT > VSHT > enamel for 1 mm thickness and dentine > VET > NKT > VEHT > VST > VSHT > enamel for 2 mm thickness.

**Table 2:** Means and standard deviations of CIE L*a*b* values of human enamel and dentine and of all ceramic materials and shades for the two different thicknesses analyzed

Material	Thickness (mm)	Color coordinates			Thickness (mm)	Color coordinates		
		L*	a*	b*		L*	a*	b*
Dentine	1	86.89 ± 3.56	− 1.22 ± 0.38^AB^	7.57 ± 2.05^abcd^	2	82.67 ± 6.70	− 0.92 ± 0.57^AB^	12.78 ± 4.99^abcdef^
Enamel	1	65.61 ± 3.21^αβγ^	− 2.35 ± 0.51	6.46 ± 2.34^aef^	2	66.54 ± 4.35^αβγ^	− 0.58 ± 1.21^ACDEF^	12.08 ± 2.34^aghijk^
NKT	1	70.29 ± 2.08	− 0.32 ± 0.48	11.00 ± 3.43^b^	2	72.40 ± 2.98	0.23 ± 0.72^C^	14.87 ± 4.05^bg^
VEHT	1	69.56 ± 2.49^α^	− 0.24 ± 0.59	9.89 ± 3.55^ce^	2	70.76 ± 3.30^α^	0.32 ± 1.03^BD^	12.83 ± 3.38^ch^
VET	1	73.31 ± 1.55	0.40 ± 0.71	12.54 ± 3.21	2	74.42 ± 2.14	1.28 ± 1.03	15.26 ± 3.19^di^
VSHT	1	67.07 ± 2.71^β^	− 1.53 ± 0.50^A^	8.46 ± 2.41^df^	2	67.86 ± 2.92^β^	− 0.10 ± 0.62^E^	10.96 ± 1.80^ej^
VST	1	69.59 ± 3.70^γ^	− 0.88 ± 0.89^B^	10.48 ± 2.57	2	70.01 ± 3.58^γ^	0.47 ± 0.96^F^	13.04 ± 2.19^fk^

*Results of statistical analysis between enamel or dentine samples and ceramic materials studied: same letter or sign within one column shows no statistical differences between those two materials

The results of one-way ANOVA based on ranked data showed highly significant differences in CIE L* mean values among dentine samples of 1 or 2 mm thickness and all the other materials, including enamel (*p* < 0.001). In the case of enamel, for a thickness of 1 mm, the most similar values of mean lightness were encountered for VSHT and VST *(p* = 1.000 and 0.116, respectively), while for a thickness of 2 mm, VSHT, VST and VEHT showed no significant differences with enamel samples of the same thickness (*p* = 1.000, *p* = 0.743 and 0.482, respectively). Besides, when the 2 thicknesses of the same tooth structure were compared with each other, no significant differences in lightness were found between enamel samples of 1 and 2 mm (*p* = 1.000) and between dentine samples of 1 and 2 mm thickness (*p* = 0.966).

Highly significant differences were identified between the mean CIE a* values of 1 mm-thick dentine and enamel samples and the other materials tested (*p* < 0.001), excepted for VSHT (*p* = 0.977) and VST (*p* = 1.000) when compared to dentine. The correlations between dental structures and ceramic materials for CIE a* values increased with the thickness of the material, showing no significant differences (*p* > 0.05) between dental structures of 2 mm thickness and the 5 materials measured, except for human dentine versus NKT, VET, VST and VSHT, and human enamel versus VET (in all cases, *p* < 0 0.001).

CIE b* values of dentine samples revealed very close mean values to enamel samples of the same thickness (*p* = 1.000). For thin samples (1 mm), both dentine and enamel samples showed significant statistical differences (*p* < 0.05) only with the less translucent materials tested (NKT, VET and VST). No statistically significant differences (*p* > 0.05) were found for the CIE b* chromatic coordinate between human enamel and 2 mm-thick dentine samples and all three ceramic systems studied.

**Fig. 4:**
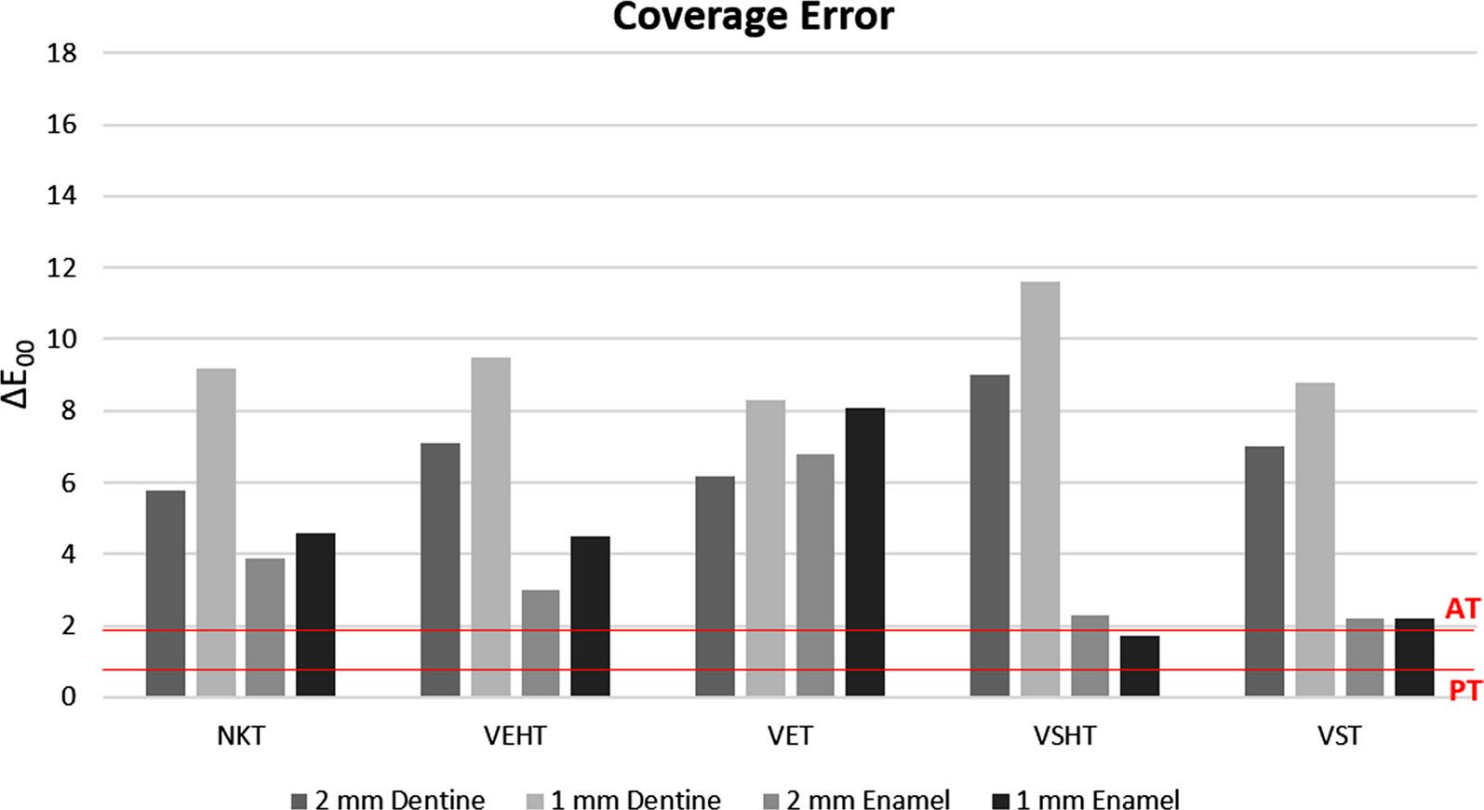
Coverage error (CE) of all studied materials for dentine and enamel obtained using ΔE_00_ color difference formula

**Fig. 5:**
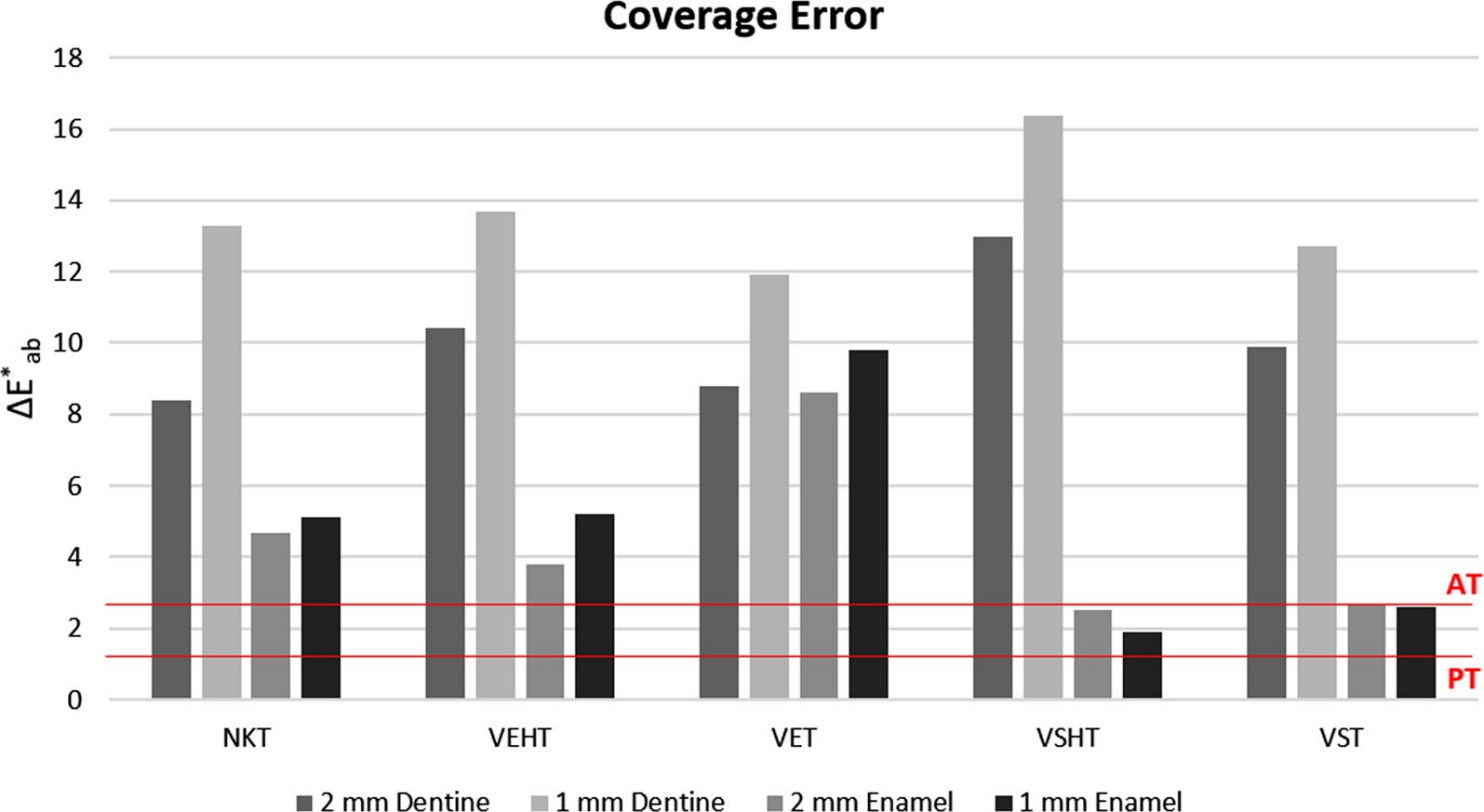
Coverage error (CE) of all studied materials for dentine and enamel obtained using ΔE^*^_ab_ color difference formula

CIELAB and CIEDE2000 Coverage Errors (CE_ab_, and CE_00_, respectively) of the different ceramic systems studied for color of dentine or enamel samples (1 and 2 mm) are represented in Figs. 4 and 5. For 2 mm dentine samples, the lowest value of CE was registered for NKT (CE_00_ = 5.8; CE_ab_ = 8.4), while for 1 mm dentine samples, the lowest CE was found for the VET system (CE_00_ = 8.3; CE_ab_ = 11.9). In all cases and for both color difference formula used, the CE values found exceeded both PT and AT. In the case of 2 mm thick enamel samples, the best CE was exhibited by the VST ceramic system (CE_00_ = 2.2) and the VSHT ceramic system (CE_ab_ = 2.5), depending on the color difference formula used for computation. For 1 mm-thick enamel samples, the VSHT systems showed the lowest CE (CE_00_ = 1.7; CE_ab_ = 1.9). The VS systems, in it´s both translucencies (VSHT and VST), showed CE values higher than PT but lower or at AT, independently of the thickness of the sample (Figs. 4, 5).

## Discussion

Nowadays, the increasing number of available all-ceramic materials leads to difficulties in choosing the proper material for the desired dental restoration. Aesthetic properties, along with composition and mechanical properties of restorative materials represent the main factors with an impact on the clinically optimal material selection. In the present study, the color coordinates of three types of all-ceramic materials with different opacities and different shades were comparatively evaluated with samples of human dentine and enamel of corresponding thicknesses. Being able to relate the properties of dental restoratives with human dental hard structures is of great importance [30], since the materials chosen for this study are supposed to render, in a biomimetical way, their reference model (which is undoubtedly the natural tooth). In this sense, all available shades and opacities of these three different categories of aesthetic ceramic materials (conventional feldspathic ceramic, machinable ZrO_2_ lithium silicate glass–ceramic and machinable hybrid ceramic) were thoroughly analysed from a colorimetric point of view.

Spectroradiometric objective color measurements are the current standard in dental research [31–35], since, unlike most of the others objective color measuring instruments that can be used in dental applications, they have the ability to provide highly accurate non-contact color readings, avoiding thus undesirable edge-loss errors characteristic to contact-type color measuring instruments. Spectroradiometric color readings of translucent samples (such as the samples analysed in the present study) will be affected by the background used for color measurements. Since the oral cavity is black, a matte opaque black ceramic tile was used as background for spectroradiometric measurements in this study.

The first null hypothesis of this study was partially rejected, as statistically significant differences in lightness between dentine samples and all the studied materials were registered, while for enamel, several materials (VSHT, VST and VEHT) showed no significant differences in CIE L* mean values when compared to 1, respectively 2 mm-thick samples. It is well known that lightness is strongly correlated to translucency [36]. A translucent material has lower lightness than an opaque material, especially when measured against a black background. This explains why VSHT and VEHT registered mean CIE L* values closer to enamel than to dentine. Surprisingly, VST, a material with a lower translucency (as presented by the manufacturer) revealed mean CIE L* values very close to enamel. This means that, in terms of lightness, Vita Suprinity in both versions (HT and T) is the best option, among studied materials, for replacement of enamel. This advantage may be due to lithium silicate crystals, which are slightly larger and more rounded, compared to those present in the feldspathic ceramic [37, 38]. The good balance between aluminium compounds (1.3%) and zirconia (15.5%) could also increase the L* values of this material, while preserving its translucent properties [37]. Furthermore, the glassy matrix of lithium silicate ceramic porcelain, where submicrometric crystallites of lithium metasilicates (Li_2_SO_3_) and lithium orthophospates (Li_3_PO_4_) are present, led to an increased translucency when compared to the two others ceramic systems [38]. Besides, VET obtained the highest L* mean values, the closest to the dentine ones. The opaqueness of this T version may be due to the higher amount of Al_2_O_3_ from its composition and to the crystal size (up to ~ 20 μm) and structure, which does not resemble to any particular mineral [14, 38]. According to the results of the present study, an increase in thickness, even with 1 mm, does not affect significantly the lightness of any of the materials tested.

It was reported that the reddish and yellowish appearance of ceramics depends on its thickness [39], similar to the findings reported in the present study, where mean measured values of CIE a* and b* color coordinates were higher for the 2 mm-thick samples than for the 1 mm ones for all ceramic systems analyzed. Similar to other studies [40], with an increase in thickness, the values of CIE a* coordinate shifted towards more reddish for all the materials and dental structures measured. This might be due to a higher concentration of pigments in greater thicknesses. In terms of similarity with dentine and enamel samples, for CIE a* color coordinate, the high translucent zirconia reinforced lithium silicate glass–ceramic (VSHT) turned out to be the best option for dentine in both thicknesses and for 2 mm-thick enamel samples. Furthermore, when analyzing the CIE b* coordinate, the ceramics with higher translucency (VSHT and VEHT) had a tendency to more blueish, with lower values than the other ceramic materials tested, but similar to enamel and close enough to dentine of both thicknesses and with no statistical significant differences between them. With an increase in thickness, the chromatic differences between the dental hard structures and the ceramic materials studied are disappearing, as the materials became more chromatic.

Although 1 and 2 mm-thick specimens are both clinically relevant as well as frequently used in dental research when studying color and optical properties of tooth structures (enamel or dentin) and dental materials (composites, ceramics, etc.) [23, 41–43], further studies including samples with different thicknesses of these all-ceramic materials should be performed in order to fully understand the relationship between shade and thickness and its influence on the final optical outcome [36]. Furthermore, a larger range of enamel and dentin samples from different types of teeth and age groups should be performed in order to confirm the present results.

Although CE is used in dentistry [26–29] mainly to assess color representation of dental shade guides, its implementation approach can be easily extended to test the color compatibility between dental ceramic systems comprising several shades and human dentine and enamel samples. Interpretation of color differences among teeth and tooth coloured materials can be done using 50:50% perceptibility (PT) and 50:50% acceptability threshold (AT) for dentistry. Recently, a consistent and systematic model for the clinical and research application and interpretation of findings related to visual thresholds was suggested [44] and is based on 50:50% PT of ΔE_00_ = 0.8 or ΔE^*^_ab_ = 1.2 and 50:50% AT of ΔE_00_ = 1.8 or ΔE^*^_ab_ = 2.7, as reported in a multi-center study [25]. A realistic goal for a ceramic system is to achieve a CE at or below the AT for color in dentistry. That would be clinically represented in the certainty of having, among all the shades of the same ceramic system, a color (a sample) that would grant a restoration that, from the chromatic point of view, would be clinically acceptable.

None of the ceramic materials included in this study provided a CE lower than the corresponding AT for the dentine samples. However, CEs lower or at the AT threshold were found for VSHT and VST for enamel samples of both thicknesses (Fig. 5). According to these results, the VITA Suprinity system was the only type of ceramic material, among those included in this study that provided an adequate color representation of the analyzed enamel samples.

In all cases, for corresponding thickness of dentine and enamel, all ceramic systems analysed consistently provided a better CE for enamel samples than for dentine samples, as expected, since the background used for objective color measurements was black and enamel samples are highly translucent. Therefore, the second null hypothesis of this study is also partially rejected, since the CE for enamel and dentine samples of all studied materials is higher (with few exceptions) than the clinically acceptable threshold for color in dentistry. It would be of real interest to confirm these results by comparing these different types of ceramic materials with a larger number of enamel and dentine samples of different thicknesses, obtained from different types of teeth with a large range of color. Furthermore, it cannot be disregarded that natural enamel and dentin are mainly anisotropic materials while the dental ceramics are isotropic materials. This implies that the direction of the incident light or the analysis of different parts of enamel and dentin may lead to different optical properties.

From a clinical point of view and taking into account the results of the present research, machinable ZrO_2_ lithium silicate glass–ceramic in its both translucency version (T and HT) could be a proper choice for veneers and crowns where only enamel and a small amount of dentine has to be replaced. Similar to previous studies [23], Vita Enamic hybrid ceramic system proved to be best suited for the reconstruction of posterior teeth, by inlays, onlays, overlays and crowns, were a deeper amount of dentine is missing and aesthetics do not represent a priority for the patient.

## Conclusions

Color coordinates of evaluated esthetic ceramic systems were different from human dentine in almost all cases. The evaluated ZrO_2_ lithium silicate glass–ceramic (VITA Suprinity) with its 2 levels of translucency, provided lower CE values (better color compatibility) with human enamel samples.

Noritake Super Porcelain EX-3 conventional feldspathic ceramic and Vita Enamic hybrid ceramic systems were more color compatible with human dentine samples, although the CE of both ceramic systems exceeded the 50:50% acceptability threshold for both dentine thicknesses studied.

## Data Availability

The datasets used and/or analyzed during the current study are available from the corresponding author on reasonable request.

## Abbreviations

CAD/CAM: Computer-aided design/computer-aided manufacturing
CE: Coverage error
CIE: International Commission on Illumination
L*: CIE lightness
a*: CIE a* green–red chromatic coordinate
b*: CIE b* blue–yellow chromatic coordinate
VSHT: Vita Suprinity® high translucent CAD/CAM ceramic blocks
VST: Vita Suprinity® translucent CAD/CAM ceramic blocks
VEHT: Vita Enamic® high translucent CAD/CAM ceramic blocks
VET: Vita Enamic® translucent CAD/CAM ceramic blocks
NKT: Noritake super porcelain EX-3® conventional feldspathic ceramic
HT: High translucent
T: Translucent
SD: Standard deviation
PT: Perceptibility threshold
AT: Acceptability threshold
